# The effect of mode of delivery on health-related quality-of-life in mothers: a systematic review and meta-analysis

**DOI:** 10.1186/s12884-022-04473-w

**Published:** 2022-02-22

**Authors:** Kate Evans, Hannah Fraser, Olalekan Uthman, Osemeke Osokogu, Samantha Johnson, Lena Al-Khudairy

**Affiliations:** Warwick Medical School, University of Warwick, Coventry, CV4 7AL England

**Keywords:** Systematic review, Meta-analysis, Mode of delivery, Vaginal delivery, Caesarean section, Childbirth, Quality-of-life

## Abstract

**Background:**

Previous research is inconclusive on the effects of mode of delivery on maternal health-related quality-of-life (HRQoL). We conducted a systematic review and meta-analysis to assess the current evidence for associations between mode of delivery and postpartum health-related quality-of-life.

**Methods:**

Electronic databases MEDLINE ALL (OVID), Web of Science, The Cochrane Library, CINAHL and EMBASE (OVID) were searched for English written articles investigating the relationship between mode of delivery and quality-of-life published form inception to 15th October 2020. Two reviewers independently screened titles and abstracts, assessed full texts, and extracted data. Meta-analysis was conducted where possible.

**Results:**

Twenty-one studies, including 19,879 women, met the inclusion criteria. A meta-analysis of 18 studies found HRQoL scores were significantly higher for women after vaginal delivery in comparison to caesarean (emergency and elective combined) (Effect Size (ES) 0.17, 95% CI 0.01–0.25, *n* = 7665) with highest scores after assisted vaginal delivery (ES 0.21, 95% CI 0.13–0.30, *n* = 2547). Physical functioning (ES 11.18, 95% CI = 2.29–20.06, *n* = 1746), physical role (ES 13.10, 95% CI = 1.16–25.05, *n* = 1471), vitality (ES 6.31, 95% CI = 1.14–10.29, *n* = 1746) and social functioning (ES 5.69, 95% CI = 1.26–10.11, *n* = 1746) were significantly higher after vaginal delivery compared to caesarean.

**Conclusions:**

Health-related quality-of-life scores were higher for women after vaginal delivery in comparison to caesarean section. Consequently, women should be encouraged to deliver vaginally where possible. The findings of this research should be available to the relevant population to help support informed choice.

**Supplementary Information:**

The online version contains supplementary material available at 10.1186/s12884-022-04473-w.

## Background

Childbirth is a major event in a woman’s life, that for many, brings significant health problems. Following birth she must recover from these whilst learning to feed and care for her newborn [1]. Whilst puerperal changes are expected to be complete within the first 6 weeks after childbirth [2] many women experience postnatal morbidities for extended periods [3–5]. Furthermore, many of these morbidities can go undetected and untreated [4, 5]. The journey to recovery is important as it impacts on the mother’s health-related quality-of-life (HRQoL) and the health of the baby [4, 5].

HRQoL is a multidimensional measure of health from the individual’s perspective. It focuses on the physical, emotional and social impact that diseases have on individuals and accounts for their goals, expectations, standards and concerns [5–8]. HRQoL has increasing importance within healthcare for its ability to estimate well-being, the impact of disease and the cost-effectiveness of interventions [5, 7, 9]. Evidence shows HRQoL is a valid measure of maternal health [5, 10].

Mode of delivery is an influential factor on postpartum HRQoL [11, 12]. Caesarean section (CS) rates are increasing with more women requesting an elective caesarean for personal and societal reasons, yet whether such perceived benefits continue after birth is debated [13–15]. Two recent reviews have explored the effects of delivery modes on HRQoL though their findings contradict [16, 17]. One study reported that HRQoL was higher in women with vaginal delivery (VD) than CS [16], whilst another found no significant difference in HRQoL between the delivery types [17]. Methodological differences between the studies may explain the contradictory findings. To our knowledge there is no paper that reviews the effects of delivery mode on HRQoL to include non-Asian populations in any period of time. This review aims to close this literature gap by systematically reviewing published literature on the association between HRQoL in postpartum women and mode of delivery.

## Methods

The protocol for this review is registered on PROSPERO (CRD42020145090).

### Search strategy

A systematic search of the electronic databases MEDLINE ALL (OVID) (which covers the main Medline database, Medline Daily and Medline EPub Ahead of Print, In Process, In Data Reviews and Other Non-Indexed Citations, PubMed-not-Medline), Web of Science (SCI-EXPANDED, SSCI and ESCI), The Cochrane Library (Cochrane reviews and Cochrane Central Register of Controlled Trials), CINAHL and EMBASE (OVID) was conducted on 10th November 2019 and updated on 15th October 2020. No date limit was applied, and filters excluded non-English language studies as translation resources were unavailable. The search strategy included full, truncated, and MeSH terms pertaining to mode of delivery (vaginal and caesarean), childbirth, HRQoL and QoL measures (Additional file 8). Reference lists of systematic reviews and included studies were also screened for potential studies. A specialist librarian (SJ) supported the search strategy.

### Eligibility criteria

Eligible studies included full text journal articles that reported overall HRQoL scores as a primary outcome, and that specified and stratified by mode of delivery. Observational studies of any design were considered appropriate for inclusion. Validated Quality of Life (QoL) tools must have been evidenced. The eligible population included women of reproductive age (15–49 years) who had experienced vaginal or caesarean delivery, including those with other health conditions (e.g., diabetes, gestational diabetes). Studies in restricted populations (e.g., critically ill (a life-threatening process that can result in mortality or significant morbidity [18])) were excluded as they are not representative of the general population.

### Study selection and data extraction

Titles and abstracts were independently reviewed by two authors (KE, HF/OO). All abstracts identified were retained for full-text assessment. Reasons were noted for exclusion. Any disagreements during study selection were resolved by referral to a third author (LAK). References of selected articles were reviewed to identify any other eligible studies. Two reviewers (KE, HF/ OO) independently extracted data from included studies using a piloted electronic data abstraction form (Additional file 9). Extracted data pertained to study location, study design, population, delivery mode, HRQoL tool, mean HRQoL scores, and follow-up.

### Quality appraisal

Two reviewers (KE, HF/ OO) independently undertook quality appraisal, resolving disagreements through discussion or referral to an independent reviewer (LAK). The Cochrane Risk of Bias In Non-Randomized Studies-of Interventions (ROBINS-I) tool was used to assess the quality of all studies [19]. Studies were assessed for sources of potential bias including method of recruiting participants, sample size, validated outcome measures, confounders, loss to follow-up and type of analysis.

### Data summary, synthesis and statistical analysis

Studies included were assessed using the Robin’s tool which includes seven domains using a high, low or unclear scale [19]. Random-effects meta-analyses were conducted using STATA SE Version 16.1 to calculate the standardised mean difference of overall HRQoL scores. Consistent measurement tools across studies for explanatory outcomes (component and dimension scores) allowed a mean difference analysis. Findings were presented using a forest plot. Due to lack of studies in the spontaneous vaginal delivery (SVD) group, SVD and assisted/ instrumental vaginal delivery (AVD/ IVD) were clustered into a single ‘vaginal delivery (VD)’ group. The weighted mean and normal distribution were used to produce the standard deviation of outcome measurements as if the combined group had never been divided into two [20]. Analysis of caesarean section included both elective and emergency caesarean sections resultant of the study data available. This review intended to pool data across measurement tools. However, the tools constructs were too divergent, so data were pooled according to individual tool type. Hedges g test was conducted to measure the effect size (ES) where measurement tools were different. Mean difference (unstandardized) was used to measure the effect size where measurement tools were the same. Effect sizes greater than 0 were considered to indicate greater HRQoL improvement in vaginal delivery than caesarean section. Egger’s test was used to assess publication bias. Meta-regression was used to explore heterogeneity and investigate the relationship between effect size of sample size (small study bias), study design, year of study (trend analysis) and location. Results were summarized using bubble plots. The cut-off date for assigning studies to ‘earlier’ and ‘recent’ groups for the study year subgroup analyses was 2016. This date was chosen to capture any recent effects not measured by previous reviews [16, 17]. Heterogeneity was assessed using the I^2^ statistic and considered low, moderate, and high at values of 25, 50, and 75% respectively [21].

Studies that were statistically incomparable were narratively summarized.

## Results

### Searching, sifting, and sorting

**Fig. 1:**
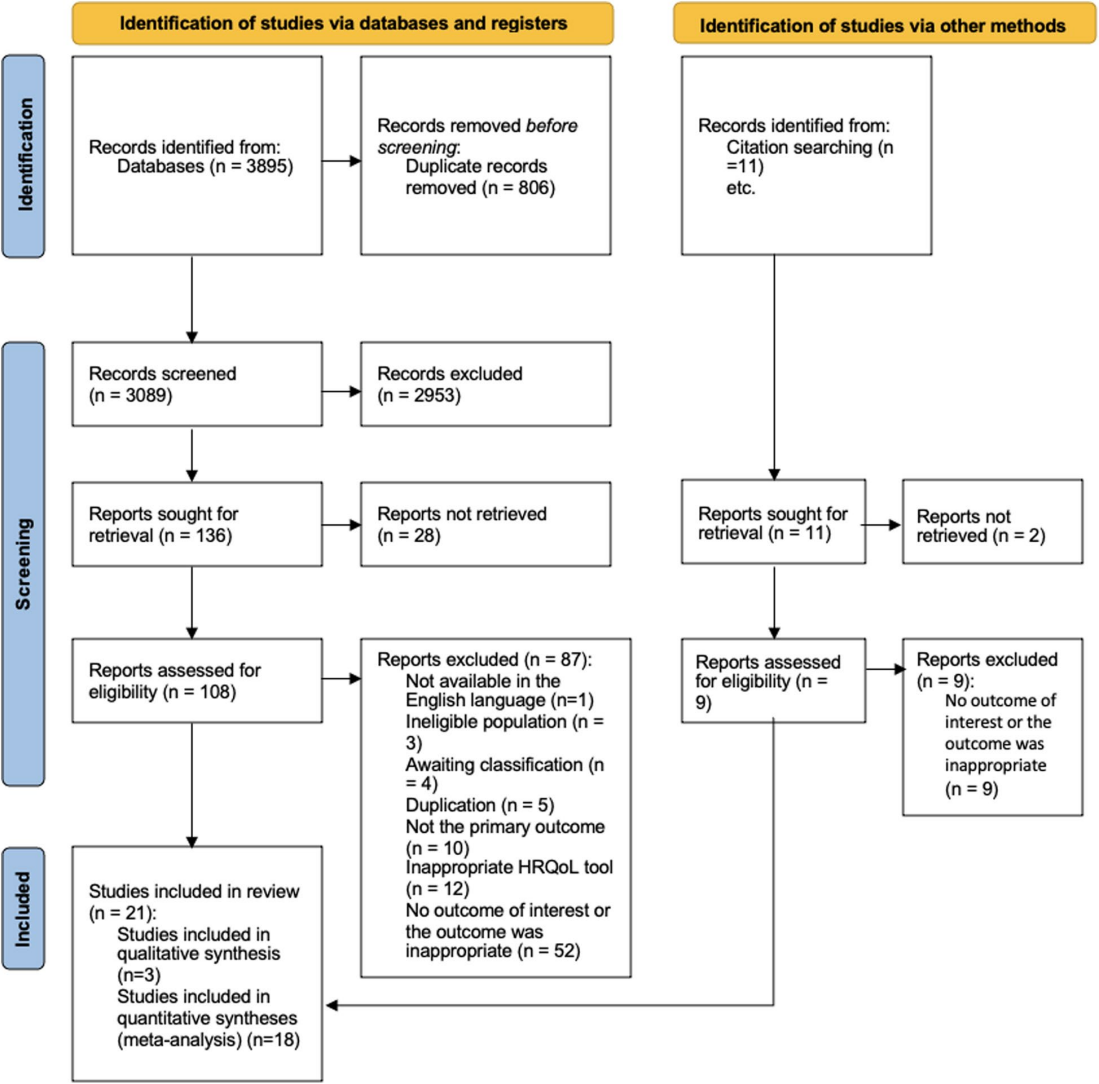
PRISMA 2020 flow diagram for new systematic reviews which included searches of databases, registers, and other sources [22]

The search of five databases identified 2501 unique records on 10th November 2019. An updated search on 15th October 2020 yielded 557 unique records. Following title and abstract sifting 146 articles were assessed for eligibility and 21 were eligible for inclusion. The main reason for exclusion was irrelevant outcomes (i.e., delivery modes not compared or QoL not reported by birth mode). Authors were contacted where information was unclear. Unavailable full text articles were excluded. The PRISMA diagram (Fig. 1) provides details of exclusions at each stage and reasons for full text exclusions can be found in Additional file 1.

### Characteristics of included studies

Twenty-one studies met the inclusion criteria and were included within the review. The studies were conducted between 2007 and 2019 across 39 countries with no particular geographical pattern. The most common locations were Bangladesh, China, Germany, Italy, India, Iran, Ireland, Spain, Turkey, and the United Kingdom (UK). Two studies were multi-country studies of which one study was conducted across 25 locations [23]. The sample size of included studies was 19,879 women. Study samples ranged from 130 to 2990 (median = 400). Timepoints for assessment of outcomes ranged between 12 h and 10 years (median = 2 months) postpartum, with diverse study durations (narrowest 12–24 h [24], widest ≤1 yr- ≥ 10 yrs. [25]). The youngest maternal age was 15 and the oldest 49 years [26, 27]. Birth modes were grouped differently. Vaginal were categorised as spontaneous (SVD, *n* = 10), assisted (AVD, *n* = 5) and vaginal (VD, *n* = 2). Caesarean sections were mostly reported as a whole group (CS, *n* = 11) although two studies reported elective (ElCS) and emergency caesarean section (EmCS) separately [24, 28]. HRQoL tools included the Medical Outcome Short Form (SF-6D,12&36, *n* = 11), European Quality of Life Five Dimension Scale (EQ-5D, *n* = 3) and World Health Organization Quality of Life (WHOQOL-BREF, *n* = 3). One study used a disease-specific measure: The Mother-Generated Index (MGI) [29]. Where authors specified validation of QoL the tools were considered accepted validated measures in this review.

Eighteen studies were meta-analysed, of which eleven provided overall HRQoL scores, four reported SF-36 summary scores: Physical Component Summary (PCS) and Mental Component Summary (MCS), and eight reported SF-36 dimensions. Three studies were narratively synthesised following due to their unique tool [30], or incomparable dimensions [27, 31]. Full characteristics are shown in Additional file 2*.*

### Risk of bias assessment

**Fig. 2:**
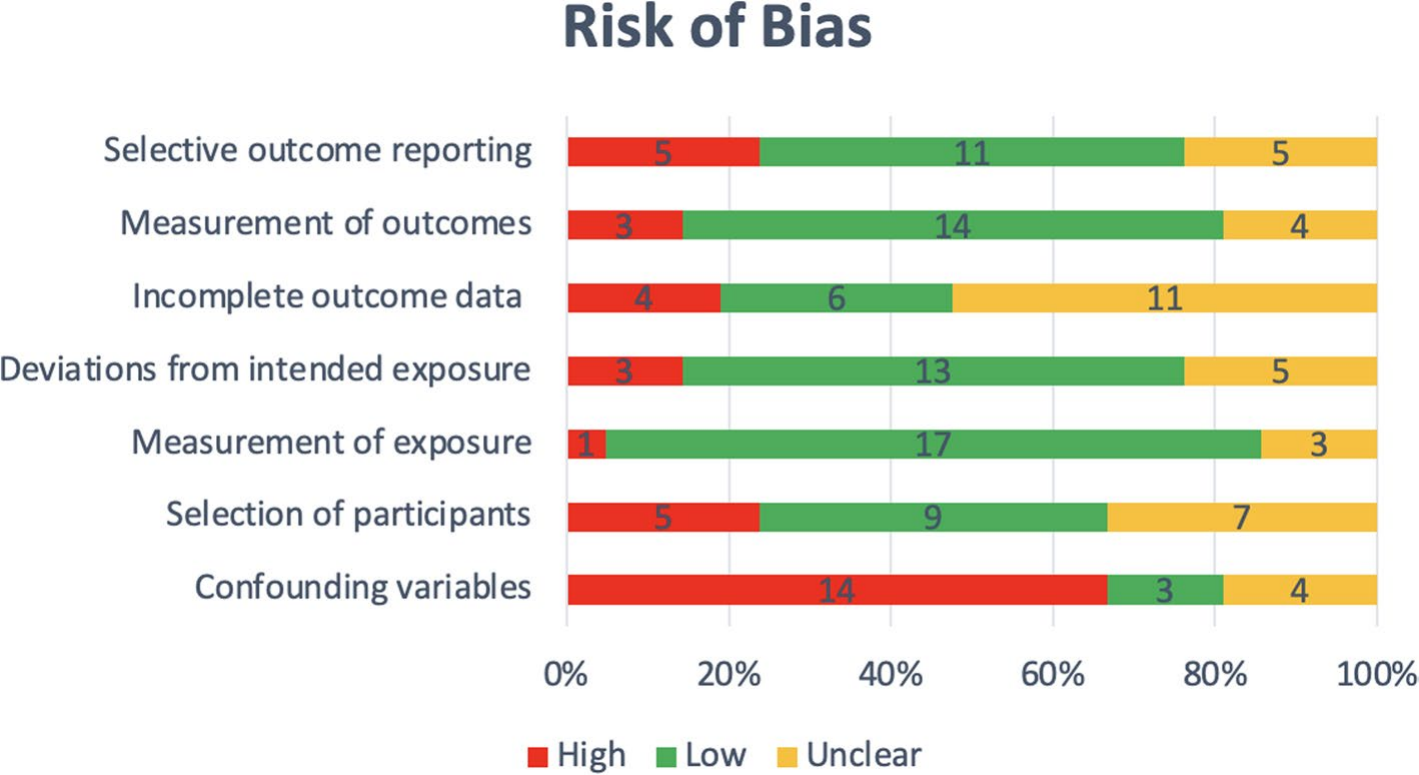
Risk of Bias criteria assessment according to the Robins tool [19]

The main limitation, effecting 67% of studies, was inadequate confirmation or a lack of consideration of confounding variables potentially leading to selection bias (Fig. 2). This is particularly an issue for studies with small sample sizes spread across multiple settings, like Kavosi et al. [32]. Studies were checked for control of the following variables: parity, labor induction, analgesics, hospital setting, preterm birth, birth weight and gestational age. Three studies adjusted for confounders outside this scope including age, education, and employment status [23, 25, 33]. One study adjusted for some, but not all, of the specified confounders listed in their analyses. Full details of the risk of bias assessment are provided in Additional file 3.

### Overall HRQoL

#### Statistical analysis of overall HRQoL scores between vaginal delivery and caesarean section

**Fig. 3:**
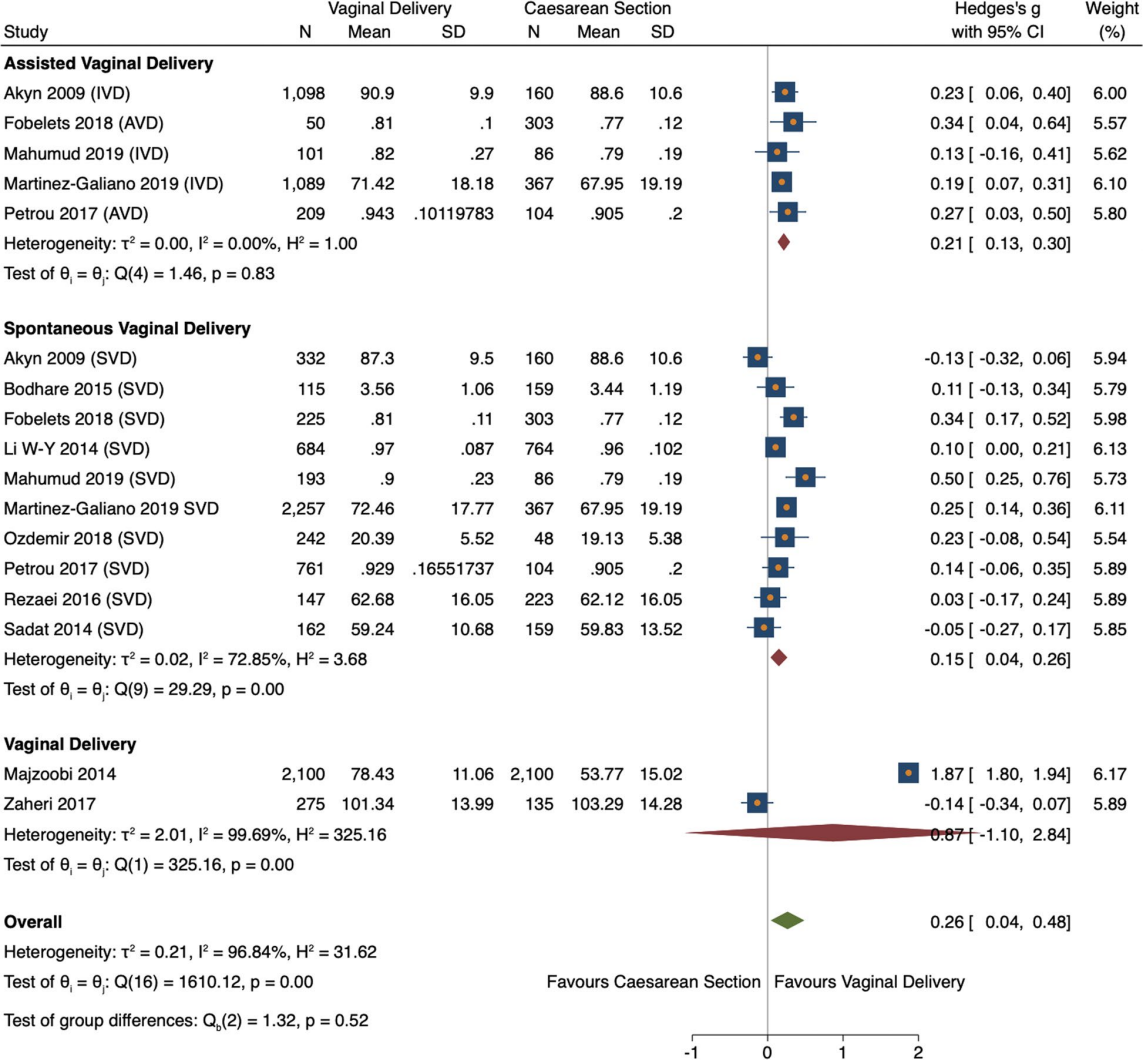
Forest plot of random effects meta-analysis of studies assessing overall HRQoL score stratifying by delivery type, outliers included

**Fig. 4:**
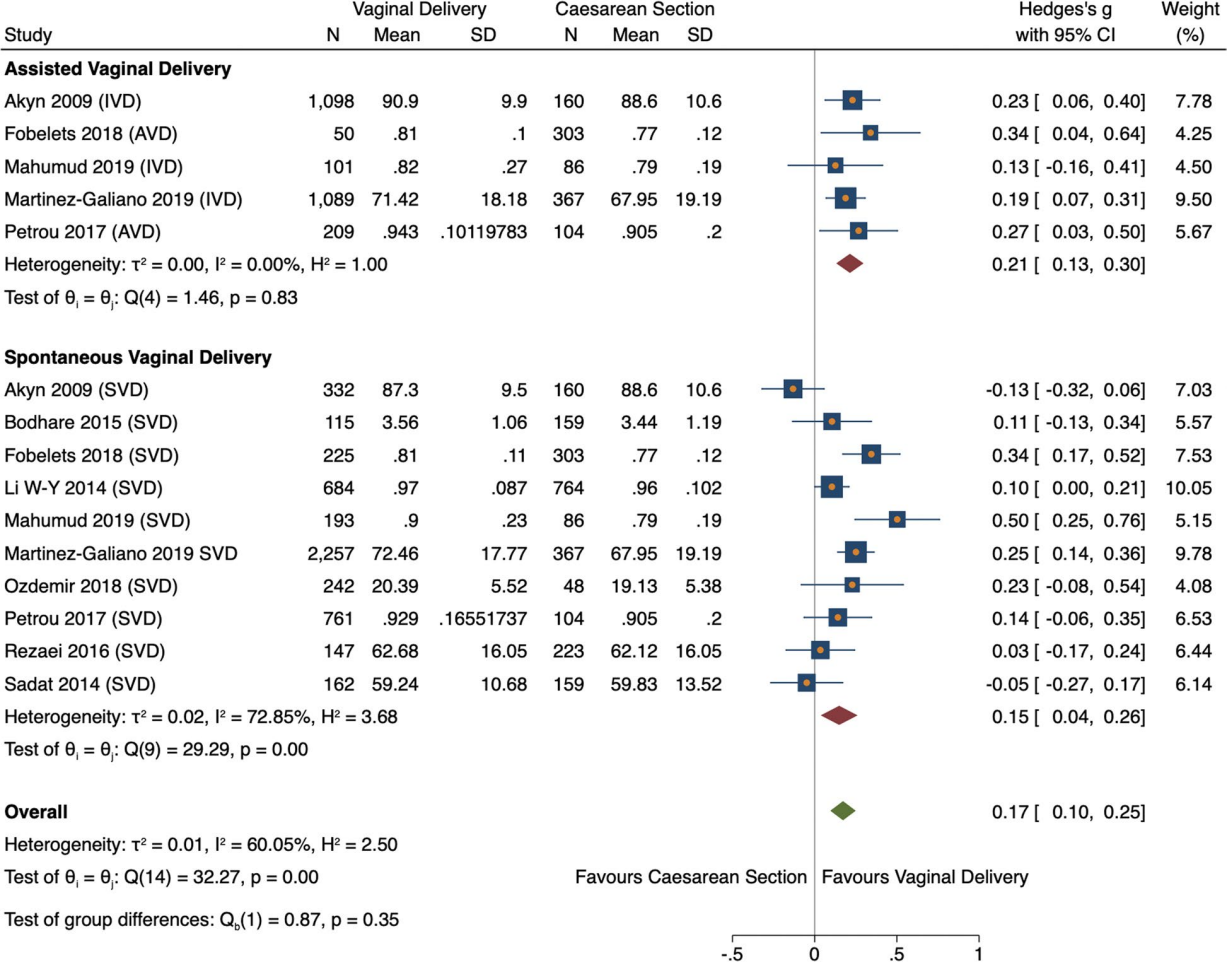
Forest plot of random effects meta-analysis of studies assessing overall HRQoL score stratifying by delivery type, outliers removed

Twelve studies were initially statistically analysed comparing overall HRQoL scores between vaginal delivery and caesarean section groups. Pooled analysis found vaginal delivery had statistically higher HRQoL than caesarean section (ES 0.26, 95% CI = 0.04–0.48, *n = 10,040, 12 studies*) (Fig. 3) [11, 26, 29, 31, 34–40]. However, the vaginal delivery group had high heterogeneity (I^2^ = 99.39%). This was caused by one study [37], whose means were considerably higher than the other studies pooled. This outlying study was removed, leaving one study remaining in the VD group thus unable to be pooled (Fig. 3) [31]. Consequently, both studies were analysed narratively [31, 37]. Ten studies were subsequently meta-analysed reporting the estimated overall standardized mean difference was statistically significant (ES 0.17, 95% CI 0.01–0.25, *n* = 7665, 10 studies) showing HRQoL was higher for VD than CS (Fig. 4) [11, 26, 29, 34–36, 38–40]. There was medium heterogeneity (60.1%) due to variation between studies rather than within-study sampling (Q = 32.27, *p* = 0.00).

#### Statistical analysis of overall HRQoL scores and vaginal delivery

Both vaginal subgroups, SVD and AVD, reported significantly higher HRQoL comparted to CS (ES 0.15, 95% CI = 0.04–0.26, *n* = *5118, 10 studies* vs ES 0.21, 95% CI 0.13–0.30, *n* = *2547, 5 studies* respectively) (Fig. 4) [11, 26, 29, 34–36, 38–40]. Variation between SVD studies was high (I^2^ = 72.85%) whereas AVD studies were harmonious (I^2^ = 0.00%). A meta-regression showed SVD had a negative non-significant relationship with AVD, therefore no stratified analysis was conducted (− 0.76, 95% CI = -0.24–0.09).

#### Subgroup analysis

**Fig. 5:**
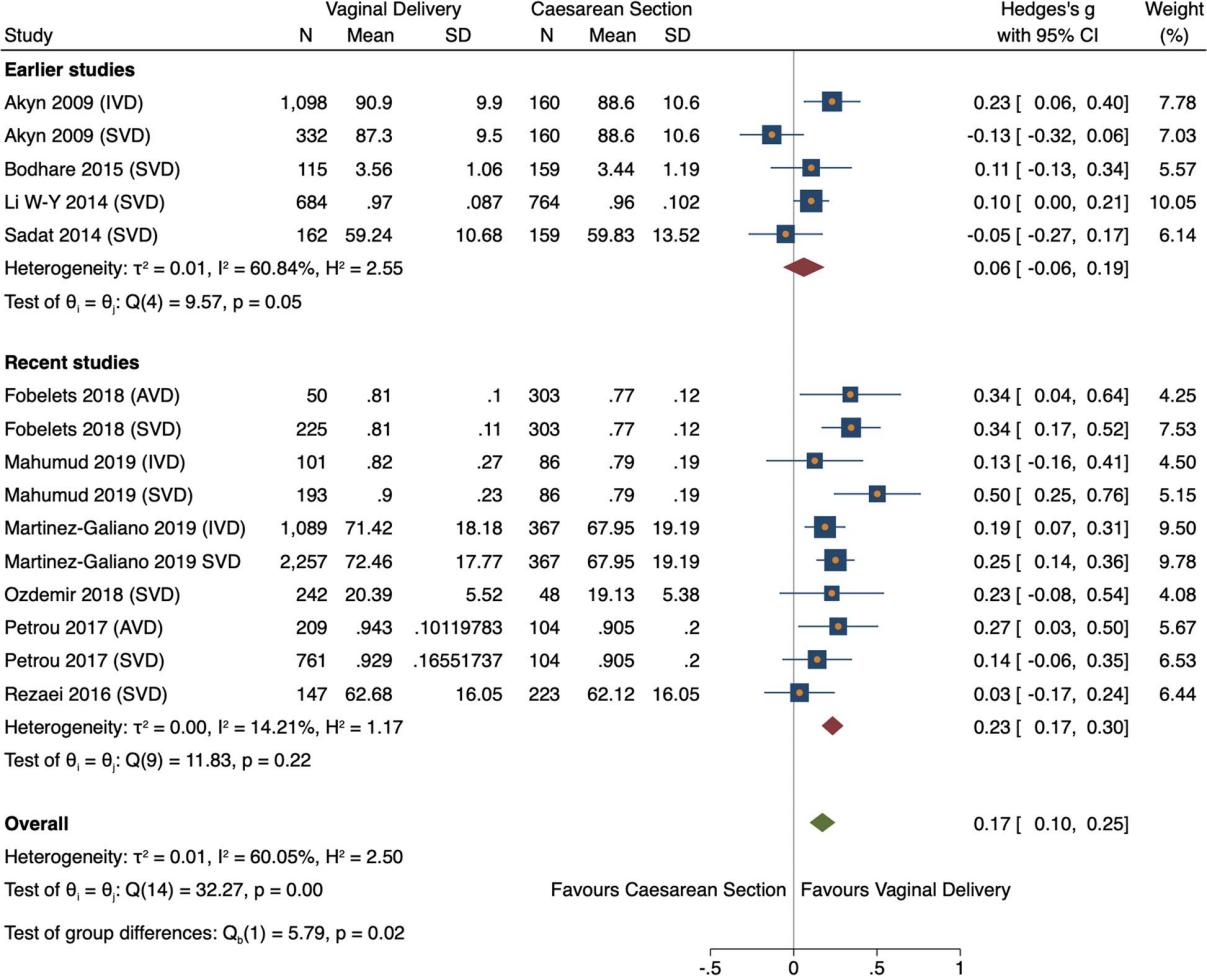
Forest plot of random effects meta-analysis of studies assessing overall HRQoL score stratified by earlier and recent studies

**Fig. 6:**
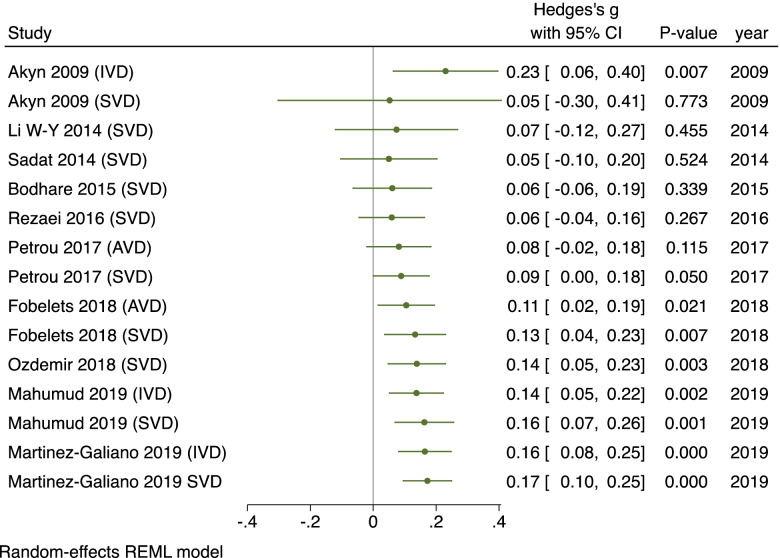
Forest plot studies assessing overall HRQoL score reporting effect size stratified by date of study

To investigate heterogeneity subgroup analyses on study year, sample size, design and location was conducted. Results showed a significant association between delivery mode and HRQoL from 2016 onwards (ES 0.23, 95% CI = 0.17–0.30, *n = 5274, 6 studies*) [11, 34, 36, 38, 39, 41], but not prior (ES 0.06, 95% CI = -0.06–0.19, *n = 3291*, *4 studies*) [26, 29, 35, 40] (Fig. 5). Meta-regression confirmed a small increase in effect in recent studies (0.02, *p* = 0.01). Chronological analysis revealed study results have been consistent since 2009, almost identical since 2018 showing that HRQoL has been significantly higher for women who delivered vaginally for over a decade (Fig. 6).

***Sample size*** had no direct impact on effect size (0.00, *p* = 0.66) evidencing results were not inflated and there was evidence of publication bias (0.55, *p* = 0.67).

##### Study design

**Fig. 7:**
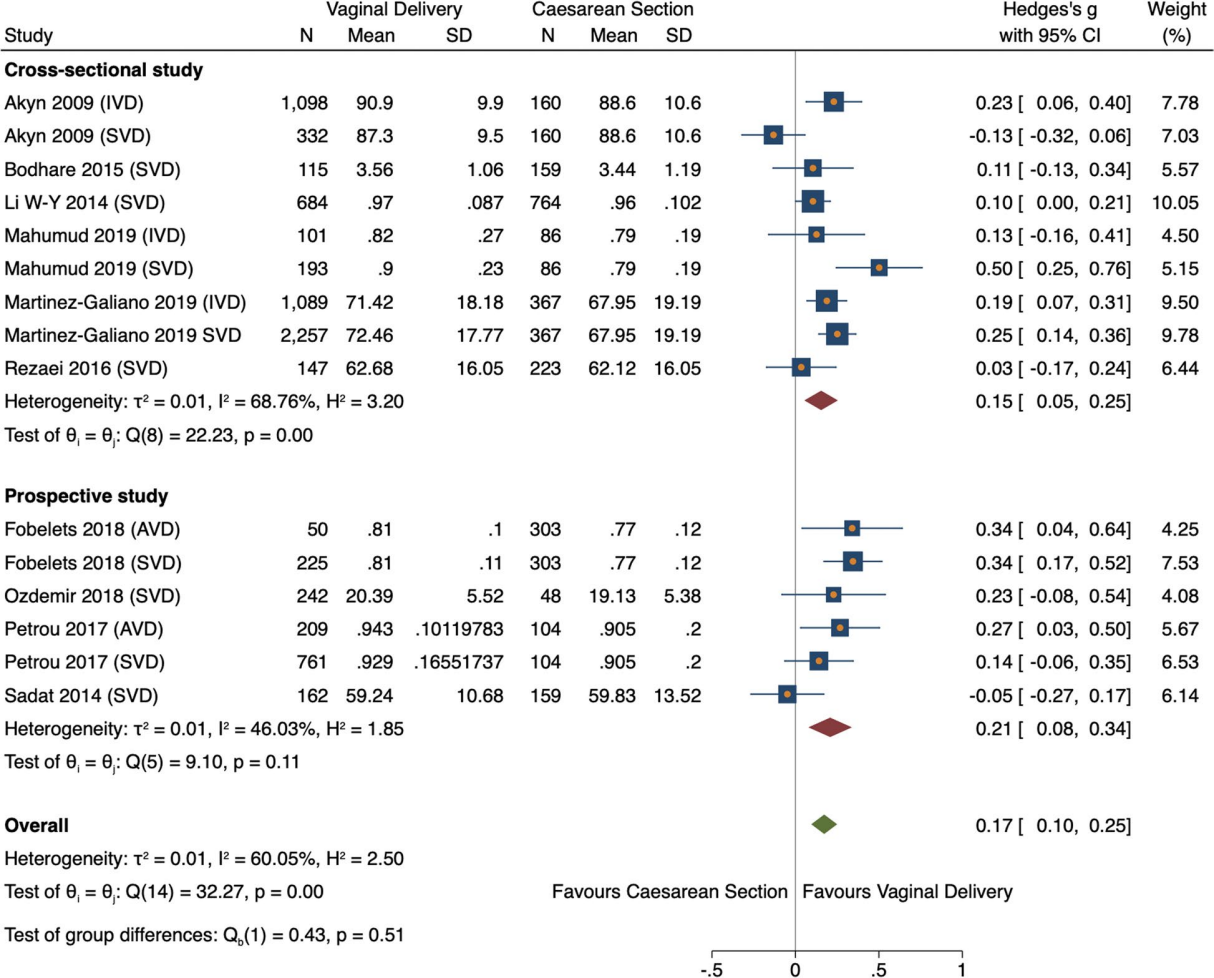
Meta-analysis forest plot of overall HRQoL total score showing subgroup analysis by study design

The effect size was higher in prospective studies (ES 0.21, 95% CI = 0.08–0.34, *n* = *1649, 4 studies*) [11, 28, 39, 42] than cross-sectional studies (ES 0.15, 95% CI = 0.05–0.25, *n* = *6016, 6 studies*) [26, 29, 35, 36, 38, 41] (Fig. 7), with medium heterogeneity present in both design types (I^2^ = 46.03% and I^2^ = 68.76% respectively)*.* Study design did not significantly change the effect size (0.054, *p* = 0.519).

##### Study location

**Fig. 8:**
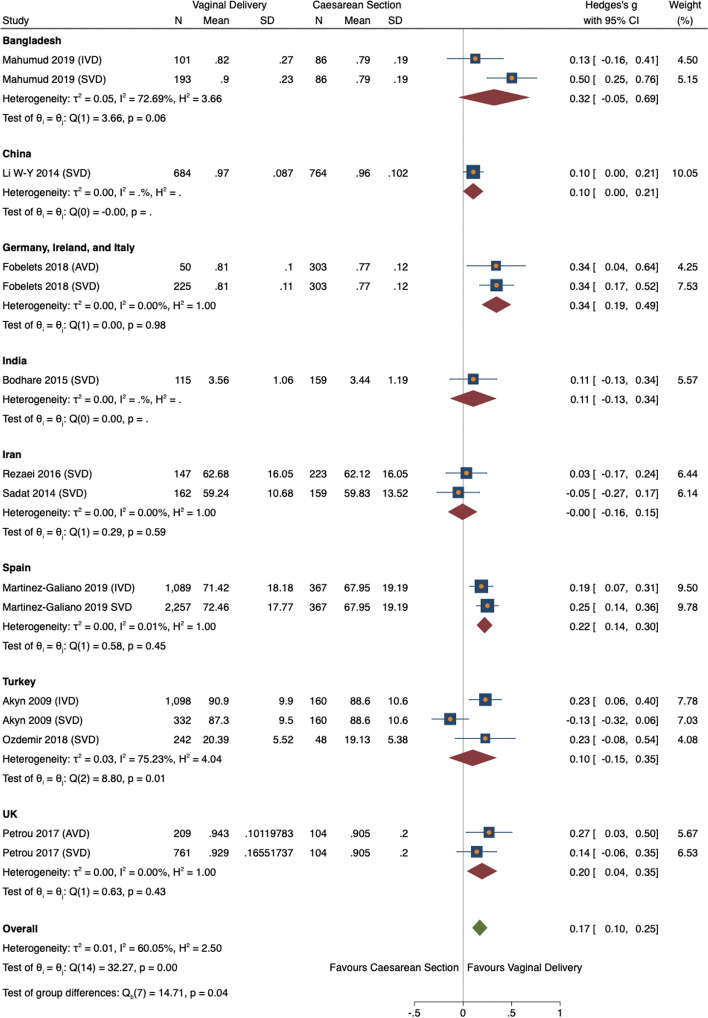
Meta-analysis forest plot of overall HRQoL score showing subgroup analysis by study location

In all locations, HRQoL was higher after VD. The association was significant in Germany, Ireland and Italy (ES 0.35, 95% CI = 0.19–0.49, *n* = *275, 1 study*) [28], Spain (ES 0.22, 95% CI = 0.14–0.30, *n* = *3346, 1 study*) [38], and the UK (ES 0.20, 95% CI = 0.04–0.35, *n* = *970, 1 study*) [38] (Fig. 8). Between study heterogeneity was medium (52.3%). Most locations had a negative, insignificant, meta-regression coefficient (*p* < 0.11) showing no influence on effect size. Germany, Ireland, and Italy had a positive, non-significant coefficient (0.016, *p* = 0.924, 95% CI = -0.30–0.33) suggestive of a positive impact on the effect size. Iran had a significantly negative coefficient (− 0.33, *p* = 0.04, 95% CI -0.65- -0.02) indicating a negative influence on the effect size. However, both statistics lack power and further research is required to confirm these results. Due to the small number of studies for all locations these results are for visual representation and should be interpreted with caution.

### Mental (MSC) and Physical Component Scores (PCS)

#### Statistical analysis of component scores (MCS and PCS) and HRQoL

**Fig. 9:**
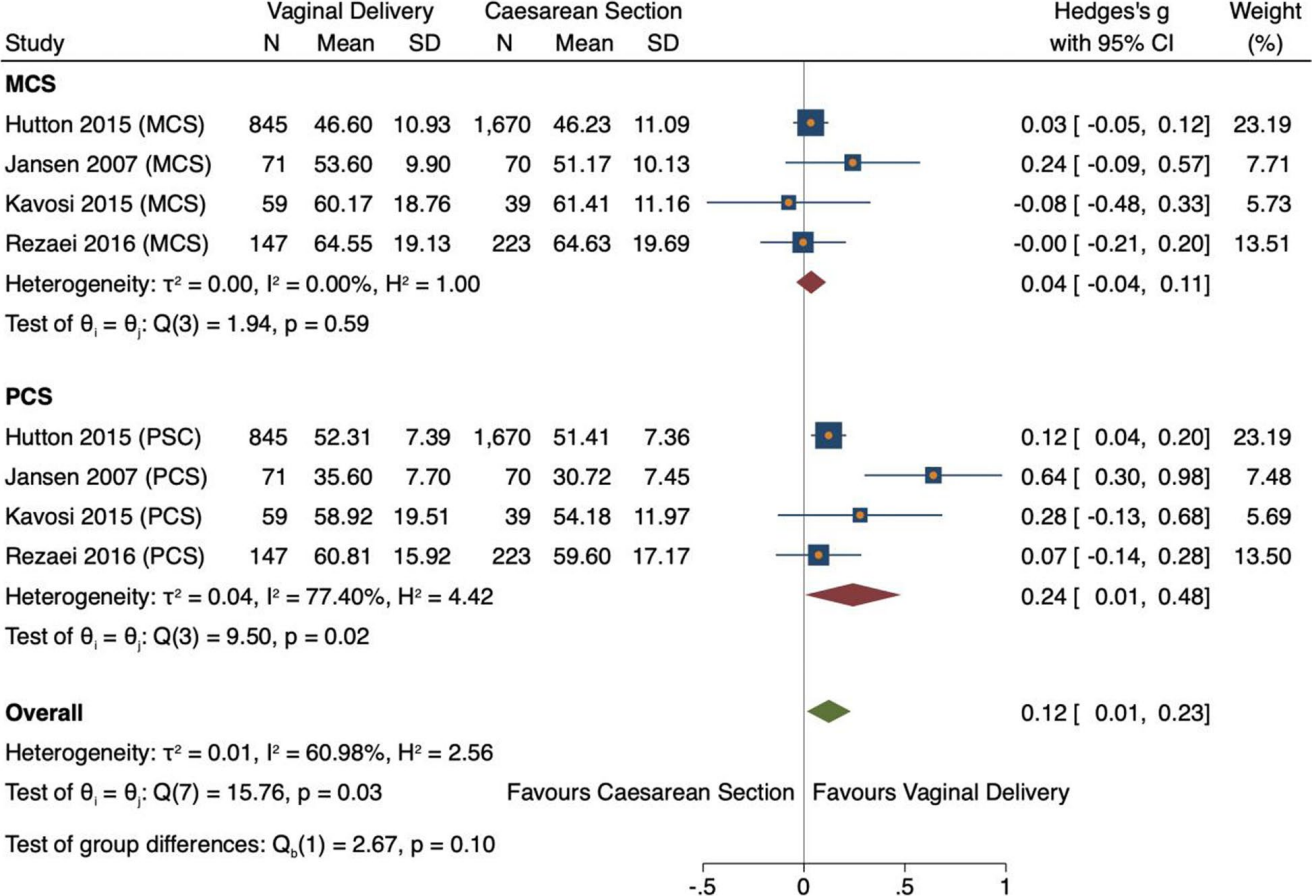
Forest plot of random effects meta-analysis of studies assessing overall HRQoL score stratified by MCS and PCS

Four studies reporting MSC and PCS scores were statistically analysed. For both components, HRQoL was found to be higher after VD than after CS, yet the association was only significant for PCS (ES 0.24, 95% CI = 0.01, 0.48, *n* = *1122, 4 studies* and ES 0.04, 95% CI = -0.04, 0.1, *n* = *1122, 4 studies* respectively) [23, 24, 32, 41] (Fig. 9). Overall, there was a small significant effect size between the birth modes and HRQoL (ES 0.12, 95% CI = 0.01, 0.23, *n* = *2244, 4 studies*) [23, 24, 32, 41]. Heterogeneity was medium between all studies reporting component scores (I^2^ = 60.98%) and between studies reporting PCS (77.40%). This was resultant of within-study sampling error (15.76, *p* = 0.03). The variation in MCS can be explained by chance (I^2^ = 0.00%) therefore no stratified analysis was conducted.

#### Subgroup analysis

**Fig. 10:**
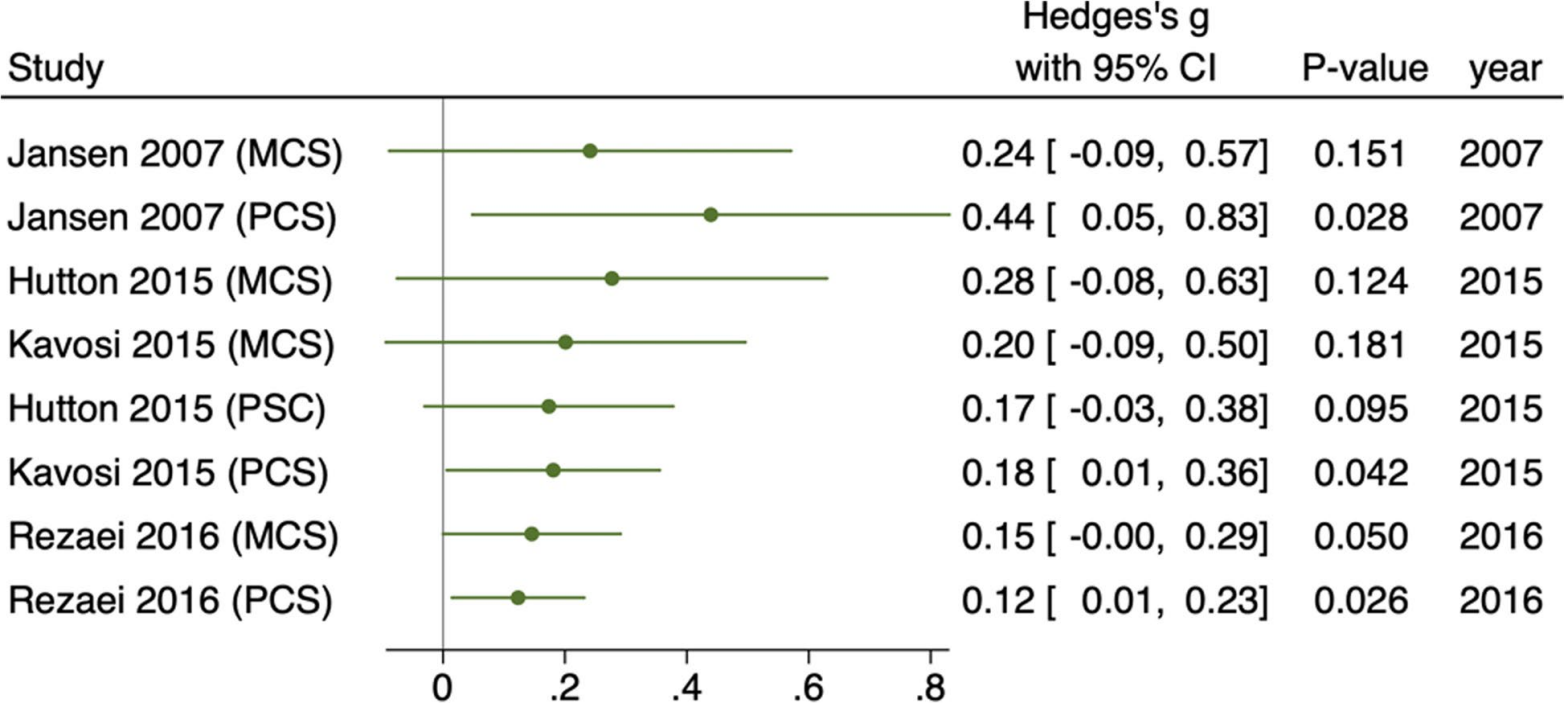
Meta-analysis forest plot showing component scores showing subgroup analysis by year of study

Findings were consistent for all studies eligible in this study regardless of date of study. Study findings have been constant since 2007, except for one PCS result in 2007 [24]. These consistent findings highlight that HRQoL has been consistently significantly higher for women who delivered vaginally for nearly 15 years (Fig. 10). The effect has marginally decreased in more recent studies (− 0.04, p0.003).

***Sample size*** showed no significant effect on heterogeneity (− 0.00, p0.42) and publication bias was not present (1.16, *p* = 0.18).

##### Study design

**Fig. 11:**
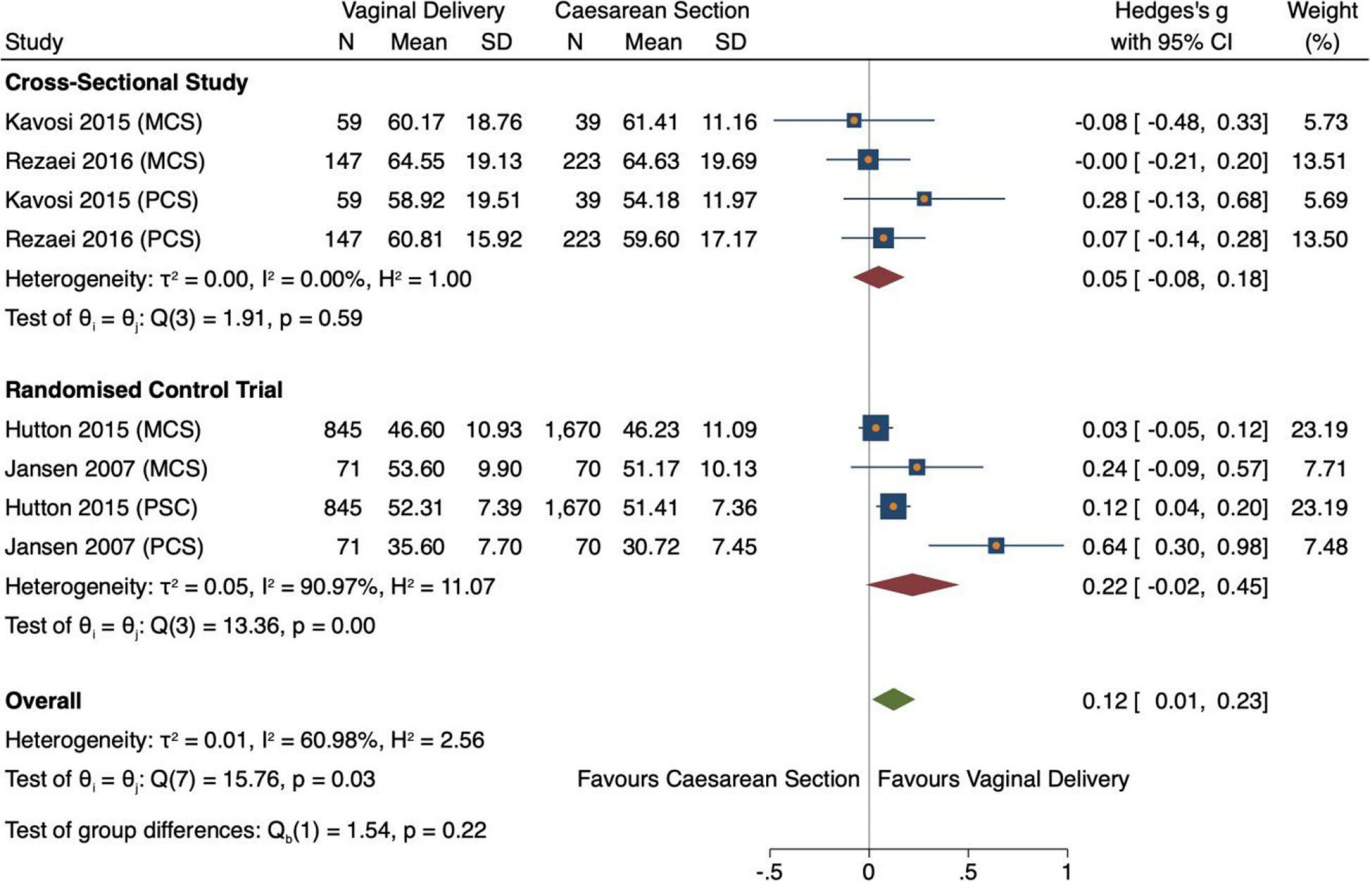
Meta-analysis forest plot showing component scores, stratified by study design

Effect size was higher, but non-significant, in RCTs (ES 0.22, 95% CI = -0.02–0.45, *n* = *1832, 2 studies)* [23, 24] (Fig. 11). Meta-regression confirmed study design had no influence on effect size (0.134, *p* = 0.32, 95% CI = -0.131–0.40) (Fig. 11). Unexpectedly heterogeneity was highest among RCTs studies (I^2^ = 90.97%), potentially caused by bias in one outlying study [24]. Subgroup analysis by location was not possible as very few studies reported location(s).

##### Study location

Subgroup analyses by location were not possible as too few studies shared locations.

### HRQoL dimensions

#### Statistical analysis of SF-36 dimensions and HRQoL

Eight studies reporting SF-36 dimensions scores were included in a statistical analysis. Vaginal delivery was associated with significantly higher physical functioning (Mean difference (MD) 11.29, 95% CI = 2.25–20.33, *n* = *1746 8 studies*) [23, 24, 28, 32, 37, 42, 43], physical role (MD 13.26, 95% CI = 1.10–25.41, *n* = *1471, 7 studies*) [23, 24, 32, 37, 42, 43], vitality (MD 6.43, 95% CI = 1.13–11.72, *n* = *1746, 8 studies*) [23, 24, 28, 32, 33, 37, 42, 43], and social functioning (MD 5.81, 95% CI = 1.31–10.32, *n* = *1746, 8 studies*) [23, 24, 28, 32, 33, 37, 42, 43] scores than caesarean section *(*Additional file 4*)*. Bodily pain, emotion role, mental health and general health had higher scores after VD, though this did not reach statistical significance. Heterogeneity was high on all dimensions (95–99%) therefore subgroup analyses were conducted to identify the cause.

#### Subgroup analysis of SF-36 dimensions and HRQoL

##### Study year

Study results were found consistent since 2015 *(*Additional file 5*)*. HRQoL was significantly higher after VD than after CS regardless of date of study conducted (MD 7.65, 95% CI = 4.77–10.53, *n = 3636, 6 studies*) [23, 24, 32, 33, 37, 40] *(*Additional file 6*).* This may be explained by the higher heterogeneity being found between the earlier studies (I^2^ = 99.52%). Meta-regression confirmed study year was not a significant cause of heterogeneity (− 0.42, *p* = 0.35).

***Sample size*** had a small significant negative effect (− 0.004, *p* = 0.00) indicating smaller studies may overestimate mean differences. Publication bias was not evident (− 0.44, *p* = 0.52).

***Study design*** had a significant impact on mean differences for cohort studies (MD 25.47, 95% CI 15.38–35.56, *n = 2378, 2 studies*) [37, 44], cross-sectional studies (MD 3.93, 95% CI 1.77–6.09, *n = 334, 4 studies*) [32, 33, 42, 43], and prospective studies (MD 7.35, 95% CI 3.98–10.73, *n = 346, 2 studies)* [24, 28] *(*Additional file 7*)*. This was confirmed by meta-regression (0.97, *p* = 0.00). No statistical difference was found between the groups (VD and CS) in randomised control trial’s (MD 0.41, 95% CI -0.02,0.84, *n = 864, 1 study*), however data was only taken from one study and therefore more data is needed to substantiate these findings.

##### Study location

**Fig. 12:**
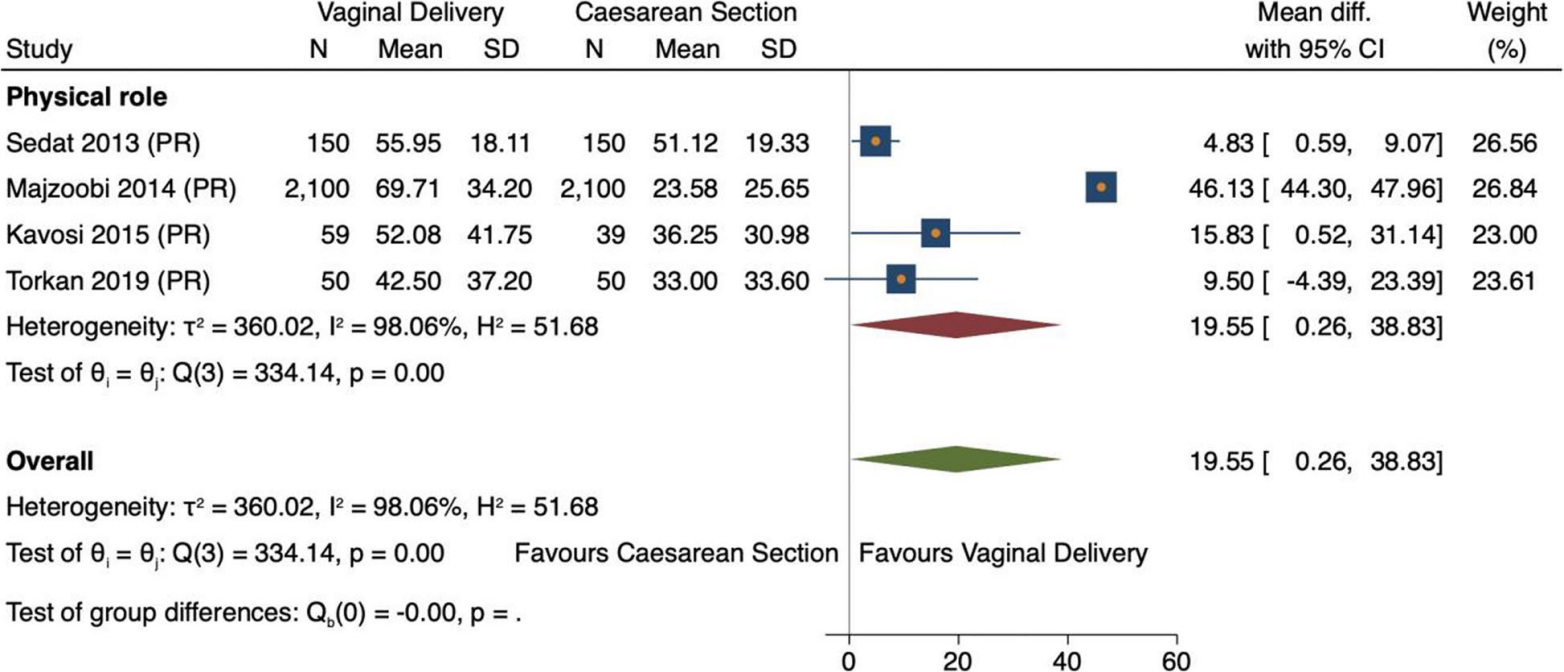
Meta-analysis forest plot showing subgroup analysis of physical role SF-36-dimension scores of Iranian studies

Subgroup analysis by location was only possible in Iran where there were multiple studies. Results found a significant impact on effect size only for physical role (MD 19.55, 95% CS 0.26–38.83, *n = 2359, 4 studies)* [32, 37, 42, 43] *(*Fig. 12*)*. This was confirmed by meta-regression (0.23, *p* = 0.05)*.*

### Narrative analysis

Three studies were narratively analysed. Two Iranian studies reported conflicting findings [37] [31]. HRQoL was significantly higher after VD in a retrospective cohort of 2100 women [37], whereas a cross-sectional study of 410 women found no significant differences between delivery modes (*p* = 0.43) [31]. This may be explained by the different timepoints observed (1 year, 6 months respectively), variation of QoL measures (SF-36, WHOQoL-BREF respectively), and small sample sizes. One Canadian study found global WHOQOL-BREF dimension scores were higher 8 weeks after vaginal delivery than after caesarean section (*n* = 181) [27]. These studies were of good quality and low bias. However, more studies are needed to substantiate these results particularly considering the small sample size.

This review was not able to conduct analyses on ethnicity and health condition, as proposed in the protocol, due to lack of information provided by the authors.

## Discussion

The global rate of caesarean sections has risen 21% in 15 years (2000–2015) [45]. More women are requesting caesarean sections for personal reasons such as fear of labor or negative vaginal delivery experiences accounting for up to 42% of all caesarean sections [46, 47]. Literature shows the impact delivery has on postpartum HRQoL varies between the different modes of delivery [11, 12, 16, 17, 48]. However, there is no unanimity in the direction of these associations as demonstrated by two reviews on the topic that had conflicting findings [16, 17]. Furthermore, these reviews were subject to selection bias caused by limiting searches by study date and language. Consequently, the included population was largely from Iran thus the findings may not generalise to non-Asian populations. The aim of this systematic review was to examine the relationship between mode of delivery and postpartum HRQoL though a systematic review of published evidence. To our knowledge this is the largest and most recent review investigating this association. The findings are expected to increase health professional’s awareness to support women’s quality-of-life after childbirth.

The results showed women who delivered vaginally had a significantly higher postpartum HRQoL than those that delivered via caesarean section. These findings are consistent with primary studies that found QoL was better after vaginal delivery in early postpartum months and 5 years later [43, 44]. They also support the findings of a related review where, up to 2015, caesarean section was negatively associated with HRQoL [16]. The current review provides new evidence that the association continues into the present. Yet not all research agrees, some found caesarean section was not a contributing factor to reduced QoL and others found no statistically significant difference between delivery types [17, 49, 50]. The discordance between literature may be explained by the diverse study methodologies observed by this review, including QoL measurement tools, HRQoL dimensions, and study location.

This review showed the strength of association varied between spontaneous vaginal delivery and assisted vaginal delivery. Both had significantly higher HRQoL scores than those who delivered by caesarean section, though there was no statistically significant relationship between the vaginal delivery types. The effect was strongest for AVD and findings harmonious. However, when interpretating these results it should be considered AVD sample size was half that of SVD.

This study revealed physical HRQoL was also better after vaginal delivery. Significant relationships were found between delivery modes and physical component, functioning, and role, with the latter two having the strongest associations of all dimensions. Furthermore, bodily pain, general health, and environmental scores were also higher after VD but non-significantly. This strengthens previous evidence that physical recovery is slower after caesarean [51]. Mental HRQoL was also found higher after VD on all dimensions with a significant association for vitality and social functioning, but non-significant for mental component, emotional role, or mental health.

This review highlights that HRQoL has been found higher after vaginal delivery, than caesarean, for over a decade. Consequently, this study provides evidence useful for the counselling of patients in obstetric practices. Health authorities are urged to disseminate this information to policy makers, clinicians and women planning a birth to ensure evidence-based decisions are fostered, providing interventions or training where necessary. Public health programmes, such as antenatal classes could be used to effectively translate this knowledge.

### Policy implications

The findings of this review have implications for obstetric practitioners and health professionals in health centers. Understanding how delivery modes effect postpartum QoL can lead to more informed choices and postpartum care, thus improving QoL. Traditional physical postpartum checks should be supplemented with QoL assessment to identify the full range of needs required for recovery after birth enabling women to reach and maintain optimum health for themselves, and their babies. Therefore findings of this study should also be used by health authorities to implement effective health programs, stratagies and policies.

This study is timely with the rise in CS rates and has implications for healthcare policy makers. The results are consistent with studies reporting that CS leads to slower recovery, prolonged hospital stays, and increased health service costs [51]. For these economic reasons policies should be reviewed to encourage women to give birth vaginally in the absence of medical indications.

### Strengths and limitations

There are several caveats in this study which can be attributed to a lack of specification of the study population. For instance, most of the delivery mode subgroups (SVD, AVD, VD, EmCS, ElCS) were analysed according to their overarching groups (VD and CS). Analysing subgroups in this combined manner induces interpretation bias. The included studies observed deliveries in hospitals and health centres. Other settings such as home births were not reported resulting in subject bias. Predominantly the studies did not adequately adjust for confounders therefore the exploration of additional factors as intended in the protocol (CRD42020145090) was restricted. Finally, data were typically collected within 6 months postpartum limiting the reviews’ ability to make longitudinal conclusions.

Review methodology limitations (first) include the exclusion criteria. Papers not presented in the English language were excluded, thus the generalizability of results is limited. Secondly qualitative studies were excluded as they did not measure HRQoL however further qualitative work would help give greater detail about women’s perceptions of their postpartum mental and physical health. Moreover, the included HRQoL tools measure different constructs thus the subscales were too different to harmonize and study results were pooled individually by tool rather than together as originally intended. However, most included studies used the SF-36 tool providing a consistent basis to this study. The SF-36 is reportedly the most suitable tool for measuring HRQoL providing the findings of this review with credibility [30, 52]. Finally, due to the different timepoints observed between studies the latest time-points were used for analysis in this study. Thus, observations of changes in QoL over time could not be made. Medium-high heterogeneity was present, and location and study design showed a small effect on dimension scores. Further research is required to substantiate these indications.

The strengths of this review include following Cochrane methods and a comprehensive search of the literature. All aspects of the reviewing process were conducted by two independent reviewers thus the likelihood of reviewing bias is low. This is an up-to-date review including the largest sample and worldwide, high-quality studies. The included QoL tools were validated and comprised of generic and disease-specifics measures. Study results were found constant since 2007 and 2014 for component and dimensions scores respectively. Meta regressions were conducted to account for heterogeneities and control for follow-up showing heterogeneity was not consequential of publication bias, study design, or year.

## Conclusion

This review showed that for all mental and physical aspects women had higher HRQoL scores after vaginal delivery after caesarean section. The association was statistically significant for physical component, physical functioning, physical role, vitality and social functioning. It is therefore recommended that pregnant women should be encouraged to deliver vaginally in the absence of medical indications.

## Supplementary Information

**Additional file 1: Supplemenaty Table T1.** Excluded studies after full text data extraction and reasons.

**Additional file 2: Supplementary Table T2.** Characteristics of Included Studies.

**Additional file 3: Supplementary Table T3.** Risk of Bias assessment score of included studies.

**Additional file 4: Supplementary Figure F1.** Forest plot of random effects meta-analysis of studies assessing overall HRQoL score, stratified by SF-36 dimensions.

**Additional file 5: Supplementary Figure 2.** Meta-analysis forest plot showing component scores showing subgroup analysis by year of study and SF-36 dimension.

**Additional file 6: Supplementary Figure 3.** Forest plot of random effects meta-analysis of studies assessing HRQoL by SF-36 dimension score, stratified by earlier and recent studies.

**Additional file 7: Supplementary Figure 4.** Meta-analysis forest plot of SF-36 dimension score, stratified by study design.

**Additional file 8.** MEDLINE Search Strategy.

**Additional file 9.** Data Extraction Form Template.

## Data Availability

The dataset(s) supporting the conclusions of this article are included within the article and included in Additional files 1, 2, 3, 4, 5 and 6.
