# Supplementary figures and images for: The effect of mode of delivery on health-related quality-of-life in mothers: a systematic review and meta-analysis
Source: BMC Pregnancy Childbirth. 2022 Feb 22;22:149. doi: 10.1186/s12884-022-04473-w (PMC8864819; doi:10.1186/s12884-022-04473-w)

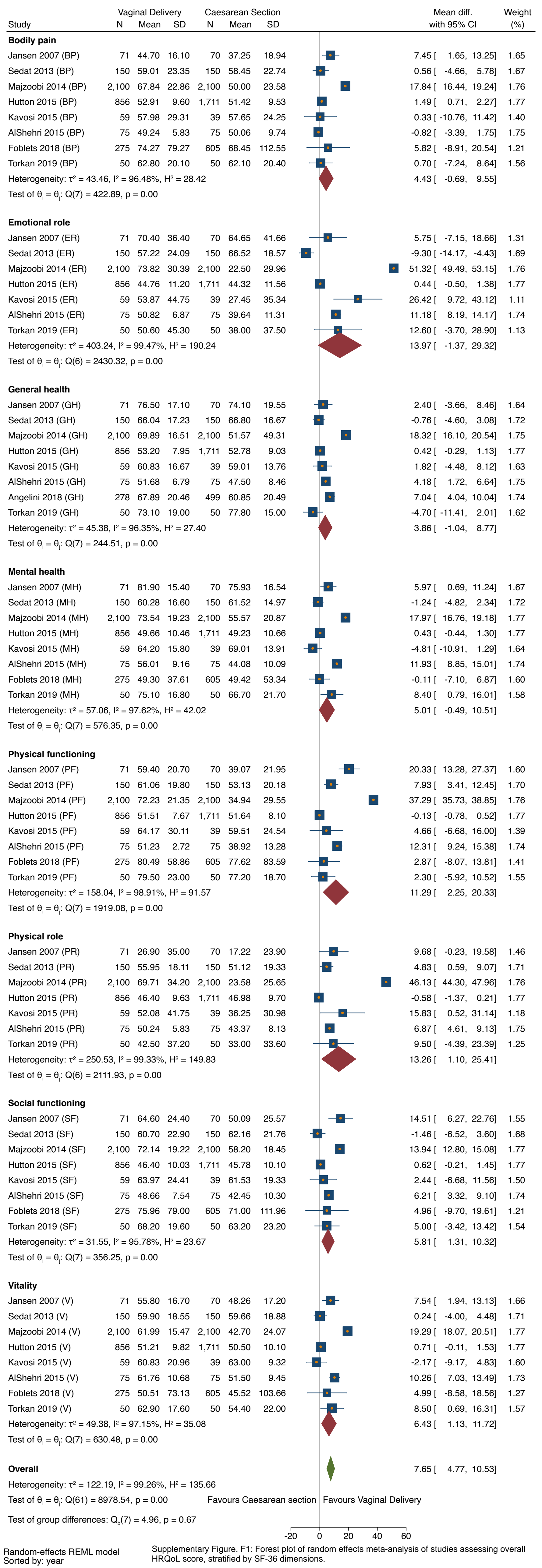

Supplement: Supplementary file 4 — Additional file 4: Supplementary Figure F1. Forest plot of random effects meta-analysis of studies assessing overall HRQoL score, stratified by SF-36 dimensions. [file 12884_2022_4473_MOESM4_ESM.pdf]

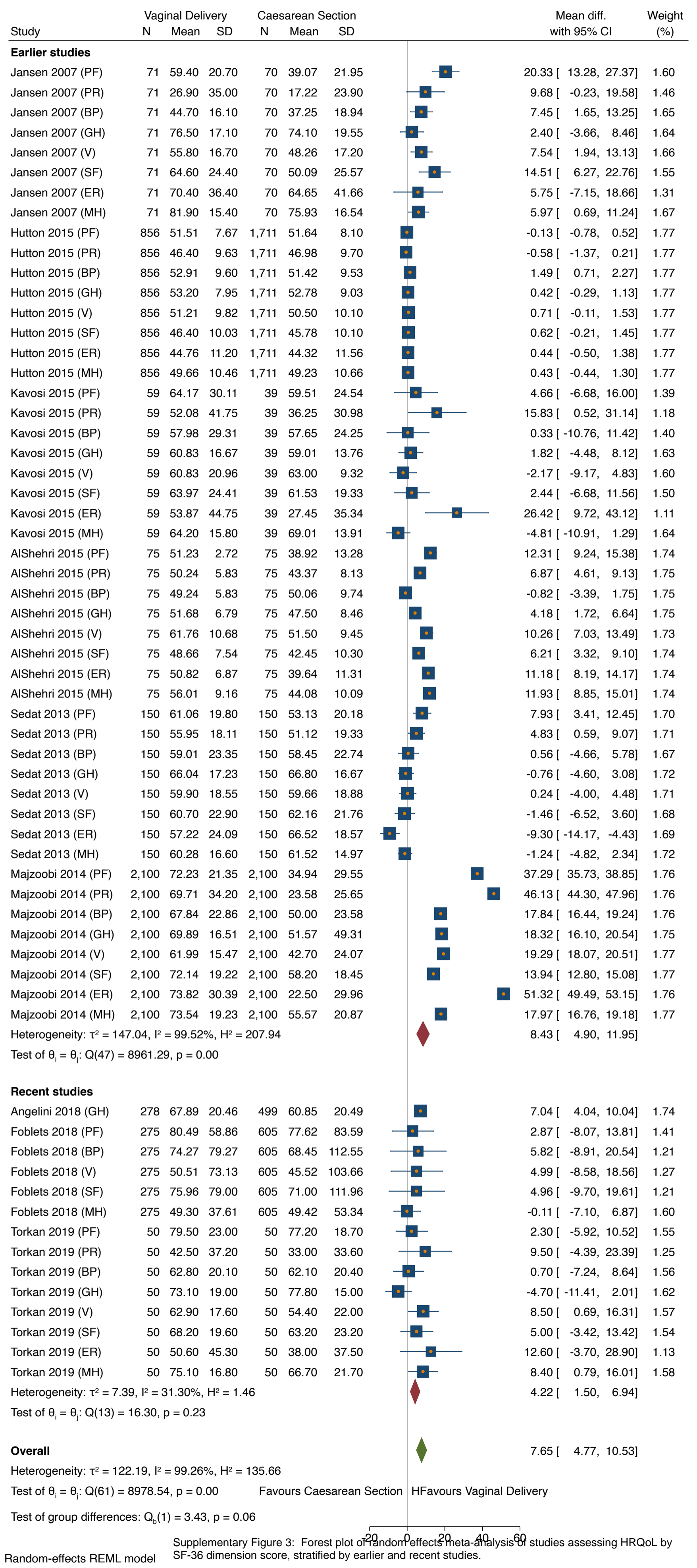

Supplement: Supplementary file 6 — Additional file 6: Supplementary Figure 3. Forest plot of random effects meta-analysis of studies assessing HRQoL by SF-36 dimension score, stratified by earlier and recent studies. [file 12884_2022_4473_MOESM6_ESM.pdf]

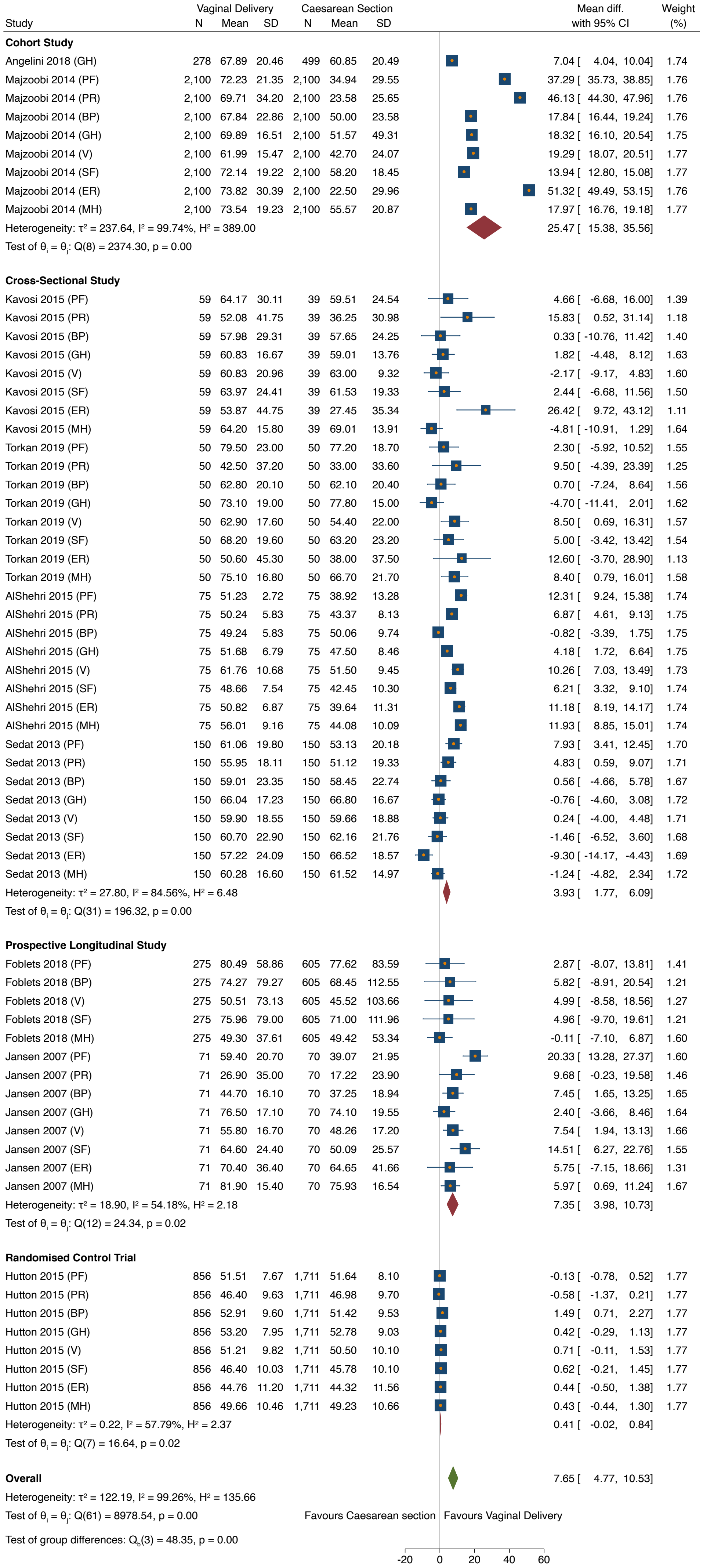

Supplement: Supplementary file 7 — Additional file 7: Supplementary Figure 4. Meta-analysis forest plot of SF-36 dimension score, stratified by study design. [file 12884_2022_4473_MOESM7_ESM.pdf]
