# Supplementary material for: The effect of mode of delivery on health-related quality-of-life in mothers: a systematic review and meta-analysis
Source: BMC Pregnancy Childbirth. 2022 Feb 22;22:149. doi: 10.1186/s12884-022-04473-w (PMC8864819; doi:10.1186/s12884-022-04473-w)
Supplement: Supplementary file 5 — Additional file 5: Supplementary Figure 2. Meta-analysis forest plot showing component scores showing subgroup analysis by year of study and SF-36 dimension. [file 12884_2022_4473_MOESM5_ESM.pdf]

Supplementary Figure 2: Meta-analysis forest plot showing component scores showing subgroup analysis by year of study and SF-36 dimension.

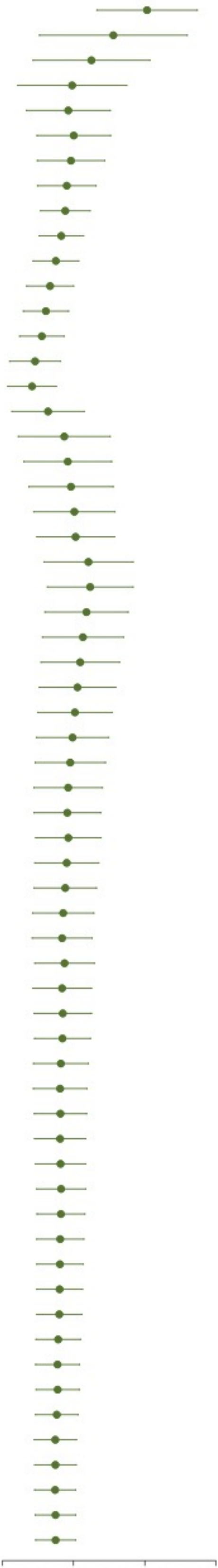

Study	Mean Diff. with 95% CI	P-value	year
Jansen 2007 (PF)	20.33 [13.28, 27.37]	0.000	2007
Jansen 2007 (PR)	15.59 [5.22, 25.97]	0.003	2007
Jansen 2007 (BP)	12.52 [4.33, 20.72]	0.003	2007
Jansen 2007 (GH)	9.82 [2.17, 17.47]	0.012	2007
Jansen 2007 (V)	9.28 [3.39, 15.16]	0.002	2007
Jansen 2007 (SF)	10.04 [4.87, 15.21]	0.000	2007
Jansen 2007 (ER)	9.65 [4.93, 14.38]	0.000	2007
Jansen 2007 (MH)	9.07 [4.97, 13.17]	0.000	2007
Sedat 2013 (PF)	8.86 [5.38, 12.33]	0.000	2013
Sedat 2013 (PR)	8.29 [5.17, 11.41]	0.000	2013
Sedat 2013 (BP)	7.53 [4.33, 10.73]	0.000	2013
Sedat 2013 (GH)	6.72 [3.43, 10.02]	0.000	2013
Sedat 2013 (V)	6.14 [2.97, 9.31]	0.000	2013
Sedat 2013 (SF)	5.56 [2.44, 8.68]	0.000	2013
Sedat 2013 (ER)	4.62 [1.05, 8.19]	0.011	2013
Sedat 2013 (MH)	4.19 [0.79, 7.59]	0.016	2013
Majzoobi 2014 (PF)	6.44 [1.37, 11.52]	0.013	2014
Majzoobi 2014 (PR)	8.71 [2.28, 15.15]	0.008	2014
Majzoobi 2014 (BP)	9.20 [3.05, 15.36]	0.003	2014
Majzoobi 2014 (GH)	9.65 [3.74, 15.57]	0.001	2014
Majzoobi 2014 (V)	10.13 [4.43, 15.82]	0.000	2014
Majzoobi 2014 (SF)	10.30 [4.87, 15.74]	0.000	2014
Majzoobi 2014 (ER)	12.10 [5.85, 18.36]	0.000	2014
Majzoobi 2014 (MH)	12.35 [6.35, 18.35]	0.000	2014
Hutton 2015 (PF)	11.83 [5.99, 17.67]	0.000	2015
Hutton 2015 (PR)	11.33 [5.65, 17.02]	0.000	2015
Hutton 2015 (BP)	10.95 [5.44, 16.47]	0.000	2015
Hutton 2015 (GH)	10.56 [5.20, 15.92]	0.000	2015
Hutton 2015 (V)	10.21 [5.00, 15.42]	0.000	2015
Hutton 2015 (SF)	9.88 [4.81, 14.94]	0.000	2015
Hutton 2015 (ER)	9.56 [4.63, 14.49]	0.000	2015
Hutton 2015 (MH)	9.26 [4.46, 14.06]	0.000	2015
Kavosi 2015 (PF)	9.14 [4.46, 13.81]	0.000	2015
Kavosi 2015 (PR)	9.29 [4.71, 13.87]	0.000	2015
Kavosi 2015 (BP)	9.06 [4.57, 13.54]	0.000	2015
Kavosi 2015 (GH)	8.85 [4.48, 13.23]	0.000	2015
Kavosi 2015 (V)	8.56 [4.26, 12.86]	0.000	2015
Kavosi 2015 (SF)	8.41 [4.21, 12.61]	0.000	2015
Kavosi 2015 (ER)	8.75 [4.58, 12.93]	0.000	2015
Kavosi 2015 (MH)	8.41 [4.29, 12.53]	0.000	2015
AlShehri 2015 (PF)	8.51 [4.49, 12.53]	0.000	2015
AlShehri 2015 (PR)	8.46 [4.54, 12.38]	0.000	2015
AlShehri 2015 (BP)	8.23 [4.38, 12.08]	0.000	2015
AlShehri 2015 (GH)	8.13 [4.37, 11.89]	0.000	2015
AlShehri 2015 (V)	8.18 [4.50, 11.85]	0.000	2015
AlShehri 2015 (SF)	8.13 [4.54, 11.71]	0.000	2015
AlShehri 2015 (ER)	8.19 [4.68, 11.70]	0.000	2015
AlShehri 2015 (MH)	8.27 [4.83, 11.71]	0.000	2015
Angelini 2018 (GH)	8.24 [4.88, 11.60]	0.000	2018
Foblets 2018 (PF)	8.14 [4.84, 11.45]	0.000	2018
Foblets 2018 (BP)	8.11 [4.85, 11.37]	0.000	2018
Foblets 2018 (V)	8.06 [4.85, 11.27]	0.000	2018
Foblets 2018 (SF)	8.01 [4.85, 11.18]	0.000	2018
Foblets 2018 (MH)	7.86 [4.74, 10.98]	0.000	2018
Torkan 2019 (PF)	7.76 [4.69, 10.83]	0.000	2019
Torkan 2019 (PR)	7.79 [4.76, 10.81]	0.000	2019
Torkan 2019 (BP)	7.66 [4.68, 10.65]	0.000	2019
Torkan 2019 (GH)	7.45 [4.49, 10.41]	0.000	2019
Torkan 2019 (V)	7.47 [4.55, 10.38]	0.000	2019
Torkan 2019 (SF)	7.43 [4.56, 10.29]	0.000	2019
Torkan 2019 (ER)	7.48 [4.64, 10.32]	0.000	2019
Torkan 2019 (MH)	7.50 [4.70, 10.29]	0.000	2019
