# Supplementary material for: The effect of mode of delivery on health-related quality-of-life in mothers: a systematic review and meta-analysis
Source: BMC Pregnancy Childbirth. 2022 Feb 22;22:149. doi: 10.1186/s12884-022-04473-w (PMC8864819; doi:10.1186/s12884-022-04473-w)
Supplement: Supplementary file 8 — Additional file 8. MEDLINE Search Strategy. [file 12884_2022_4473_MOESM8_ESM.docx]

## Additional file 8 MEDLINE Search Strategy

**Database(s): Ovid MEDLINE(R) ALL**1946 to October 15, 2020

| [**#**](https://ovidsp.dc1.ovid.com/ovid-b/ovidweb.cgi?&S=MKCHFPBOKBACOKCMKPPJPFKIDDPAAA00&Sort+Sets=descending) | **Searches** | **Results** |
| --- | --- | --- |
| 1 | exp Delivery, Obstetric/ | 161023 |
| 2 | parturition.mp. or exp Parturition/ | 45386 |
| 3 | exp Cesarean Section/ and vaginal delivery.mp. | 19812 |
| 4 | childbirth.mp. | 39868 |
| 5 | exp Postpartum Period/ | 74282 |
| 6 | 1 or 2 or 3 or 4 or 5 | 287015 |
| 7 | exp "Quality of Life"/ | 557613 |
| 8 | HRQoL.mp. | 33014 |
| 9 | health-related quality of life.mp. | 78917 |
| 10 | eq-5d.mp. | 19139 |
| 11 | euroqol.mp. | 11088 |
| 12 | sf-36.mp. | 38860 |
| 13 | sf-6d.mp. | 1668 |
| 14 | sf-12.mp. | 8829 |
| 15 | 7 or 8 or 9 or 10 or 11 or 12 or 13 or 14 | 587670 |
| 16 | 6 and 15 | 2849 |
| 17 | limit 16 to (english language and humans) | 2600 |
| 18 | limit 17 to yr=2019-20201015 | 513 |
| 19 | limit 18 to yr=2019-20201015 | 173 |
