# Supplementary material for: The effect of mode of delivery on health-related quality-of-life in mothers: a systematic review and meta-analysis
Source: BMC Pregnancy Childbirth. 2022 Feb 22;22:149. doi: 10.1186/s12884-022-04473-w (PMC8864819; doi:10.1186/s12884-022-04473-w)
Supplement: Supplementary file 9 — Additional file 9. Data Extraction Form Template. [file 12884_2022_4473_MOESM9_ESM.docx]

## Additional file 9 Data Extraction Form Template

Data Extraction Form

General Study Information

| Initials of person extracting data | KE |
| --- | --- |
| Study ID *(endnote number)* |  |
| Study citation/reference |  |
| Reference of other reports of this study (e.g protocol, offshoot publication) |  |

Study Characteristics

**Methods**

|  | **Descriptions as stated in report/paper** | **Location in text or source** *(pg & ¶/fig/table/other)* |
| --- | --- | --- |
| Aim of study |  |  |
| Design |  |  |
| Duration |  |  |
| Inclusion criteria |  |  |
| Exclusion criteria |  |  |
| Study funding sources |  |  |

**Participants**

|  | Description  *Include comparative information for each intervention or comparison group if available* | Location in text or source *(pg & ¶/fig/table/other)* |  |
| --- | --- | --- | --- |
| Country |  |  |  |
| Setting *(hospital/home)* |  |  |  |
| Sample size |  |  |  |
| Method of recruitment of participants *(e.g. phone, mail, clinic patients)* |  |  |  |
| Demographic characteristics | \| **Maternal:** \| **Yes** \| **No** \| **Unclear** \| **If yes, detail** \| \| --- \| --- \| --- \| --- \| --- \| \| Age  *Mean (SD)* \|  \|  \|  \|  \| \| BMI  *Mean (SD)* \|  \|  \|  \|  \| \| Ethnicity % \|  \|  \|  \|  \| \| Education status \|  \|  \|  \|  \| \| Marital civil status \|  \|  \|  \|  \| \| Socioeconomic status \|  \|  \|  \|  \| \| Parity \|  \|  \|  \|  \| \| Mode of delivery \|  \|  \|  \|  \| \| Years since delivery \|  \|  \|  \|  \| \| Gestational age \|  \|  \|  \|  \| \| Others \|  \| \| \| \| |  |  |
| Baseline imbalances |  |  |  |
| Withdrawals and exclusions | \|  \| **Yes** \| **No** \| **Unclear** \| **If yes, detail** \| \| --- \| --- \| --- \| --- \| --- \| \| Flow chart \|  \|  \|  \|  \|  \|  \| Description \| \| --- \| --- \| \| No. approached/invited \|  \| \| No. eligible \|  \| \| No. started/included in study \|  \| \| People excluded from the study, number and reason(s) \|  \| \| No. at end \|  \| \| % followed-up \|  \| |  |  |
| Notes: | | | |

**Outcomes**

|  | Description as stated in report/paper | Location in text or source *(pg & ¶/fig/table/other)* |
| --- | --- | --- |
| Primary Outcome |  |  |
| Secondary Outcome |  |  |
| Power |  |  |
| Type of HRQoL tool |  |  |

| HRQoL score | Vaginal delivery:   \|  \| **Yes** \| **No** \| **Unclear** \| **Detail** \| \| --- \| --- \| --- \| --- \| --- \| \| Overall HRQOL average \|  \|  \|  \|  \| \| Average by physical component score (PCS) and mental component score (MSC) \|  \|  \|  \|  \| \| Average HRQoL by dimension *e.g. Overall QoL, Physical function, Physical role, Bodily pain, General health, Vitality, Social role functioning, Emotional role, Mental health.* \|  \|  \|  \|  \|   Caesarean section:   \|  \| **Yes** \| **No** \| **Unclear** \| **Detail** \| \| --- \| --- \| --- \| --- \| --- \| \| Overall HRQOL average \|  \|  \|  \|  \| \| Average by physical component score (PCS) and mental component score (MSC) \|  \|  \|  \|  \| \| Average HRQoL by dimension *e.g. Overall QoL, Physical function, Physical role, Bodily pain, General health, Vitality, Social role functioning, Emotional role, Mental health.* \|  \|  \|  \|  \| |  |
| --- | --- | --- | --- | --- | --- | --- | --- | --- | --- | --- | --- | --- | --- | --- | --- | --- | --- | --- | --- | --- | --- | --- | --- | --- | --- | --- | --- | --- | --- | --- | --- | --- | --- | --- | --- | --- | --- | --- | --- | --- | --- | --- |
| Time points measurement *(e.g. No. mean ±SD at baseline, 3 months, 6 months etc)* |  |  |
| Notes *(any data transformation, e.g. SE to SD etc.)*: | | |

**Analysis**

|  | Description as stated in report/paper | Location in text or source *(pg & ¶/fig/table/other)* |
| --- | --- | --- |
| Method of analysis |  |  |
| Imputation of missing data *(e.g. assumptions made for ITT analysis)* | \| Yes \| No \| Unclear \| If yes, detail \| \| --- \| --- \| --- \| --- \| \|  \|  \|  \|  \| |  |
| Adjust for confounders | \|  \| Yes \| No \| Unclear \| If yes, detail \| \| --- \| --- \| --- \| --- \| --- \| \| Parity \|  \|  \|  \|  \| \| Gestational age at delivery \|  \|  \|  \|  \| \| Induction of labor \|  \|  \|  \|  \| \| Analgesics \|  \|  \|  \|  \| \| Hospital setting \|  \|  \|  \|  \| \| Preterm birth \|  \|  \|  \|  \| \| Birth weight \|  \|  \|  \|  \| |  |
| Notes: | | |

**Risk of Bias**

| **Domain** | **Risk of bias** | | | **Support for judgement**  *(include direct quotes where available with explanatory comments)* | **Location in text or source** *(pg & ¶/fig/table/other)* |
| --- | --- | --- | --- | --- | --- |
|  | Low | High | Unclear |  |  |
| **Confounding variables:**  *Selection bias caused by the inadequate confirmation and consideration of*  *confounding variable* |  |  |  |  |  |
| **Selection of participants:**  *Selection bias caused by the inadequate selection of participants* |  |  |  |  |  |
| **Measurement of exposure:**  *Performance bias caused by the inadequate measurement of exposure* |  |  |  |  |  |
| **Bias due to deviations from intended exposure**  *Performance bias –* *differences in care between groups* |  |  |  |  |  |
| **Incomplete outcome data**  *Attrition bias caused by the inadequate handling of incomplete outcome data* |  |  |  |  |  |
| **Bias in measurement of outcomes**  *Measurement bias* |  |  |  |  |  |
| **Selective outcome reporting?**  *Reporting bias* |  |  |  |  |  |
| Other bias |  |  |  |  |  |
| Notes: | | | | | |
